# GRP 78 antibodies are associated with clinical phenotype in neuromyelitis optica

**DOI:** 10.1002/acn3.50905

**Published:** 2019-09-30

**Authors:** Fumitaka Shimizu, Yukio Takeshita, Yuka Hamamoto, Hideaki Nishihara, Yasuteru Sano, Masaya Honda, Ryota Sato, Toshihiko Maeda, Toshiyuki Takahashi, Susumu Fujikawa, Takashi Kanda

**Affiliations:** ^1^ Department of Neurology and Clinical Neuroscience Yamaguchi University Graduate School of Medicine Ube Japan; ^2^ Department of Neurology Tohoku University Graduate School of Medicine Miyagi Japan; ^3^ Department of Neurology Yonezawa National Hospital Yamagata Japan

## Abstract

**Background:**

We previously reported the association between blood–brain barrier (BBB) dysfunction and glucose‐regulated protein 78 (GRP 78) autoantibodies in neuromyelitis optica (NMO).

**Objective:**

We clarify whether the BBB‐endothelial cell activation induced by immunoglobulin G (IgG) is associated with the clinical phenotype, disease activity, and markers of BBB disruption.

**Methods:**

We purified serum IgG from 24 serum samples from patients with NMO spectrum disorder (NMOSD), who were positive for anti‐AQP4 antibodies (longitudinally extensive transverse myelitis [LETM], *n* = 14; optic neuritis [ON], *n* = 6; other phenotype, *n* = 4) and nine healthy controls. IgG was exposed to human brain microvascular endothelial cells (TY10) and the number of nuclear NF‐*κ*B p65‐positive cells, as a marker of endothelial cell activation, was analyzed using a high‐content imaging system. Change in BBB permeability was also measured. The presence of GRP78 autoantibodies was detected by Western blotting.

**Results:**

In the LETM group, IgG significantly induced the nuclear translocation of NF‐*κ*B p65 in comparison to the ON and healthy control groups. A significant correlation was observed between the number of NF‐*κ*B nuclear‐positive cells and clinical markers of BBB disruption, including Gd enhancement in spinal MRI and the cerebrospinal fluid/serum albumin ratio. This effect was significantly reduced at the remission phase in the individual NMOSD patients. Furthermore, GRP78 antibody positivity was associated with the LETM phenotype and disease severity in NMOSD patients.

**Conclusion:**

Endothelial cell activation was associated with the LETM phenotype, clinical markers of BBB disruption and disease activity. These observations may explain the phenotypic differences between the NMOSD subtypes, LETM, and isolated ON.

## Introduction

Neuromyelitis optica (NMO) is an inflammatory disease associated with recurrent episodes of optic neuritis (ON) and longitudinally extensive transverse myelitis (LETM), leading to severe loss of visual and motor function.^1^ A specific feature of NMO is the presence of an autoantibody against aquaporin 4 (AQP4), which is densely expressed in the astrocytic foot processes; recent studies have demonstrated that anti‐AQP4 antibodies have a pathogenic role.^2^

Isolated ON is one of the major clinical signs in NMO as well as in multiple sclerosis (MS).^3^ The ON attacks in NMO are more severe than those in multiple sclerosis (MS), and can lead to unilateral or bilateral blindness.^3^ In addition, the lesions and involvement in optic chiasm and tracts in NMO are more extensive in comparison to those in MS in the MRI.^4^ Serum anti‐AQP4 antibody‐positive patients with isolated ON are recently classified as having NMO spectrum disorder (NMOSD), because they have a high risk of eventual conversion into definite NMO.^5^, ^6^

The disruption of the blood–brain barrier (BBB) plays key roles in the pathogenesis of NMOSD.^7^, ^8^, ^9^ We recently identified that glucose‐regulated protein (GRP) 78 autoantibodies are a potential biomarker associated with BBB‐endothelial cell activation in NMO.^10^ However, it remains unclear whether endothelial activation is associated with the disease activity or clinical phenotype of NMO. In the present study, we evaluated the contribution of IgG in serum samples obtained from individual patients with each clinical phenotype of NMO (ON and LETM) to BBB breakdown using human BBB‐derived immortalized endothelial cells. We next clarified the association between BBB disruption and the patients’ clinical profiles and investigated the GRP78 antibody status of patients with these two phenotypes of NMO.

## Materials and Methods

### Patient sample

This study was approved by the ethics committees of the Medical Faculties of Yamaguchi Universities. Written informed consent was obtained from each participant.

Serum samples were collected from 24 NMOSD patients who were diagnosed at Yamaguchi University Hospital based on the revised criteria for NMOSD (Table 1). All patients were found to be positive for anti‐AQP4 antibodies using either an immunofluorescence method or ELISA methods.^11^ We included the 14 samples from patients with LETM attacks (isolated LETM, *n* = 13; definite NMO, *n* = 1), six samples from patients with ON attacks (isolated ON, *n* = 5; definite NMO, *n* = 1) and four samples from patients with other attacks (short myelitis, *n* = 3; brainstem symptom, *n* = 1) during the acute phase within 1 month after the initiation of the attack (Table 1). All LETM patients showed paresis in the legs (Patients 1–14). One LETM patient (Patient 4) initially showed area postrema syndrome before developing severe quadriplegia with respiratory failure. Another patient with other NMOSD phenotype (Patient 24) presented with brainstem syndromes (double vision and dizziness), and a lesion of brainstem including area postrema was observed in the magnetic resonance imaging (MRI) (Fig. S1). We also included five samples from LETM patients in the remission phase, who were being treated with corticosteroids after relapse and who had been in clinical remission for at least 6 months (Patient 1, 2, 3, 7, and 9 in Table 1). Five isolated ON patients had first or second attack of unilateral or bilateral visual loss and showed optic nerve changes in MRI, including enlargement, T2 hyperintensity, and gadolinium enhancement; however, no abnormal brain or spinal cord lesions were observed in MRI (Patient 16, 17, 18, 19, 20). Cerebrospinal fluid (CSF) samples were concurrently obtained from all NMOSD patients during the acute phase. We investigated the disease duration, number of relapses, change in the score in the Expanded Disability Status Scale (EDSS) from before to after relapse (ΔEDSS), IgG index (as a marker of intrathecal IgG synthesis), CSF/serum albumin ratio (Q Alb) (as a marker of BBB dysfunction), length of the spinal cord lesion in MRI, and the presence of Gd‐enhanced lesions in MRI in the 24 NMOSD patients (Table 1).

**Table 1:** Clinical information of IgG from NMO patients

Pt Nos.	Disease/Attack Phenotype	Age/Sex	Relapse Number	ΔEDSS	IgG index	Q Alb	Spinal lesion (Length)	Gd‐ MRI	GRP Ab	% NFκB p65	Permeability
1	LETM	48/F	1	8.5	0.74	0.0091	16	(−)	(+)	1.22	0.433
2	LETM	27/F	1	8.5	0.55	0.0054	3	(−)	(+)	1.62	0.431
3	LETM	60/F	2	8.5	0.96	0.0076	5	(+)	(+)	2.40	0.455
4	LETM	32/M	1	9.5	0.52	0.0137	8 + BS	(+)	(+)	3.45	0.471
5	LETM	62/F	1	4	N/D	N/D	3	(−)	(+)	1.29	0.468
6	LETM	60/F	3	8.5	0.47	0.0055	3	(−)	(+)	0.60	0.473
7	LETM	57/F	4	6.5	0.55	0.0119	7	(+)	(+)	1.38	0.509
8	LETM	59/M	4	3.5	0.53	0.0060	3	(−)	(+)	1.16	0.498
9	LETM	76/F	1	9	0.1	0.0089	8＋BS	(+)	(+)	1.59	0.332
10	LETM	75/F	1	9	1.22	0.0085	5	(−)	(−)	0.78	0.349
11	LETM	69/F	1	8.5	0.47	0.0101	3	(+)	(−)	1.52	0.371
12	NMO/LETM	62/F	2	9	N/D	N/D	11	(+)	(−)	2.02	0.390
13	LETM	83/F	3	9	0.92	0.0104	6	(+)	(−)	1.90	0.247
14	LETM	72/F	2	8.5	0.76	0.0103	13	(+)	(+)	1.79	0.214
15	NMO/ON	16/F	8	5.5	1.14	0.0054	2		(−)	0.42	0.181
16	ON	65/M	2	1	0.45	0.0063	0		(−)	1.08	0.244
17	ON	67/F	1	5	0.54	0.0064	0		(−)	1.20	0.230
18	ON	65/F	2	2	0.55	0.0048	0		(−)	0.78	0.261
19	ON	56/F	1	5	0.58	0.0044	0		(+)	0.38	0.230
20	ON	74/F	1	5	0.67	0.0043	0		(−)	1.23	0.261
21	NMO/SM	66/F	12	3	0.66	0.0083	3	(−)	(−)	0.36	0.239
22	NMO/SM	31/F	3	1	0.61	N/D	5	(+)	(−)	0.301	0.245
23	NMO/SM	44/F	3	0	0.43	0.0069	3	(+)	(−)	0.11	0.355
24	BS	52/F	4	1	0.63	0.0078	0	(+)	(−)	0.20	0.371

Pt Nos, patient numbers; NMO, neuromyelitis optica; LETM, longitudinally extensive transverse myelitis; ON, optic neuritis; SE, short myelitis; BS, brainstem syndrome; M, Male; F, Female; ΔEDSS, change in the score on the Expanded Disability Status Scale (EDSS) from before to after relapse; Gd‐MRI, presence of Gd‐enhanced spinal lesions in magnetic resonance imaging (MRI); IgG index/Q Alb, immunoglobulin G (IgG) index as the marker of intrathecal IgG synthesis, CSF/serum albumin ratio (Q Alb) as a marker of BBB integrity; GRP Ab, presence of GRP78 autoantibodies in IgG from patients; %NF*κ*B p65: % of NF‐*κ*B p65 nuclear positive cells; Permeability: 10‐kDa dextran permeability of TY10 cells after exposure to NMOSD‐IgG.

In addition, nine individuals served as healthy controls (HCs) (male, *n* = 4; female, *n* = 5; mean ages, 32.6 years). All samples were immediately stored at − 80°C until the analysis and were inactivated at 56°C for 30 min immediately before the analysis. The IgG preparations were purified from sera using a Melon Gel IgG Spin Purification Kit (Thermo Fisher Scientific, MA).

### Immunohistochemistry and the high‐content imaging assay

TY10 cells are adult human brain microvascular endothelial cells (BMECs) immortalized with temperature‐sensitive SV40 large T antigen (tsA58), as previously described.^12^ All of the analyses were performed 2 days after a temperature shift from 33 to 37ºC. The cells were cultured in medium containing IgG (final concentration, 500 µg/mL) obtained from patients with NMOSD or healthy controls after substitution for serum‐free MCDB 131 medium for 1 h for the NF‐*κ*B p65 analyses; the primary Abs (NF‐*κ*B p65 rabbit monoclonal antibody [mAb]) and secondary Abs (Alexa Fluor 488 goat anti‐rabbit IgG) were previously described.

TY10 cells were fixed with 4% paraformaldehyde (PFA), washed and then permeabilized with 0.3% Triton X‐100. After blocking overnight in 5% FBS/0.3% Triton X‐100 in PBS, the cells were incubated with primary Abs, followed by the secondary Abs at room temperature.

Five thousand cells per well were plated in Greiner CELLSTAR® 96‐well plates (Greiner), then immunostaining for NF‐*κ*B p65 was performed. The plates were scanned, and images were captured by an In Cell Analyzer 2000 (GE healthcare) at × 20 magnification with six fields of view per well (equivalent to 800–1000 cell events). The images were then analyzed with the In Cell Analyzer software program (GE healthcare). The data represent the mean value of triplicate experiments.

### The paracellular permeability of 10‐kDa dextran

TY10 cells were cultured on 24‐well collagen‐coated Transwell tissue culture inserts (0.4‐mm pore size) for 3 days at 33°C and then 2 days at 37°C. TY10 cell monolayers were exposed, on the luminal side, to NMOSD IgG or control‐IgG (500 μg/mL) for 24 h at 37°C. After the replacement of the media, FITC‐dextran fluorescence (10 kDa; Sigma‐Aldrich) was added to the luminal insert (final concentration, 1 mg/mL), and 200 *μ*L of medium was collected from the abluminal chamber over 40 min. After transfer into 96‐well black plates, fluorescence signals were measured at 490/520 nm (absorption/emission) wavelengths using a FlexStation 3 Multi‐Mode microplate reader (Molecular Devices).

### Western blotting using human recombinant GRP78 protein

Western blotting was performed as previously described.^7^, ^8^ We used the human full length GRP78 recombinant protein (Abcam, MA, U.S.A) as antigen. Individual IgG (5 µg/mL) from 14 LETM patients, six ON patients, four other NMOSD phenotype patients and 10 healthy controls, and anti‐GRP78 antibodies (dilution 1:200) was used as the primary antibody. The protein samples (2 μg) were fractionated in a 10% gel and electrophoretically transferred onto polyvinylidene difluoride membranes (Amersham, Chalfont, UK), as previously described.^7^, ^8^ The membranes were treated with the primary antibody in PBS‐T and 5% milk for an hour, followed by incubation with the anti‐human secondary fluorescent antibodies (dilution 1:5000) for an hour. The bands were visualized with an enhanced chemiluminescence kit (ImmunoStar LD, Japan). The relative density of each band was measured using the Quantity One software program (Bio‐Rad, Hercules, CA).

### Depletion of GRP78 antibodies from LETM‐IgG or ON‐IgG

For immunoprecipitation, 500 μg/mL of LETM‐IgG (Patient 3) or ON‐IgG (Patient 17) was incubated with 5 μg of HEK 293T cell lysates with or without the overexpression of FLAG‐tagged GRP78 (Origene) for 4 h at 4°C, and then incubated with 40 *μ*L of anti‐FLAG‐IgG coupling resin (EZview Red Anti‐FLAG M2 Affinity Gel beads; Sigma‐Aldrich), for 2 h at 4°C. After the GRP78 antigen–antibody complexes were precipitated, the supernatants (LETM‐IgG or ON‐IgG with/without GRP78 antibodies) were used for the analysis.^10^ Images were captured and evaluated by an In Cell Analyzer 2000 (GE Healthcare) at × 20 magnification.

### Statistical analysis

All statistical analyses were performed using the Prism 7 software program (Graph Pad Software). For analyses with a single comparison, either the unpaired Mann–Whitney *U* or a paired Student’s *t*‐test was used to determine statistical significance (two‐sided). For analyses with multiple comparisons, a one‐way ANOVA between individual groups was performed using Tukey multiple comparisons test. All values are expressed as the mean ± SEM. Pearson’s correlation coefficients were used to test associations. The Fisher exact probability test was used to assess differences in the GRP78 antibody positivity between groups. *P < 0.05 was considered to indicate statistical significance.

## Results

### IgGs from the acute LETM patients induced BBB‐endothelial cell activation

**Figure 1:**
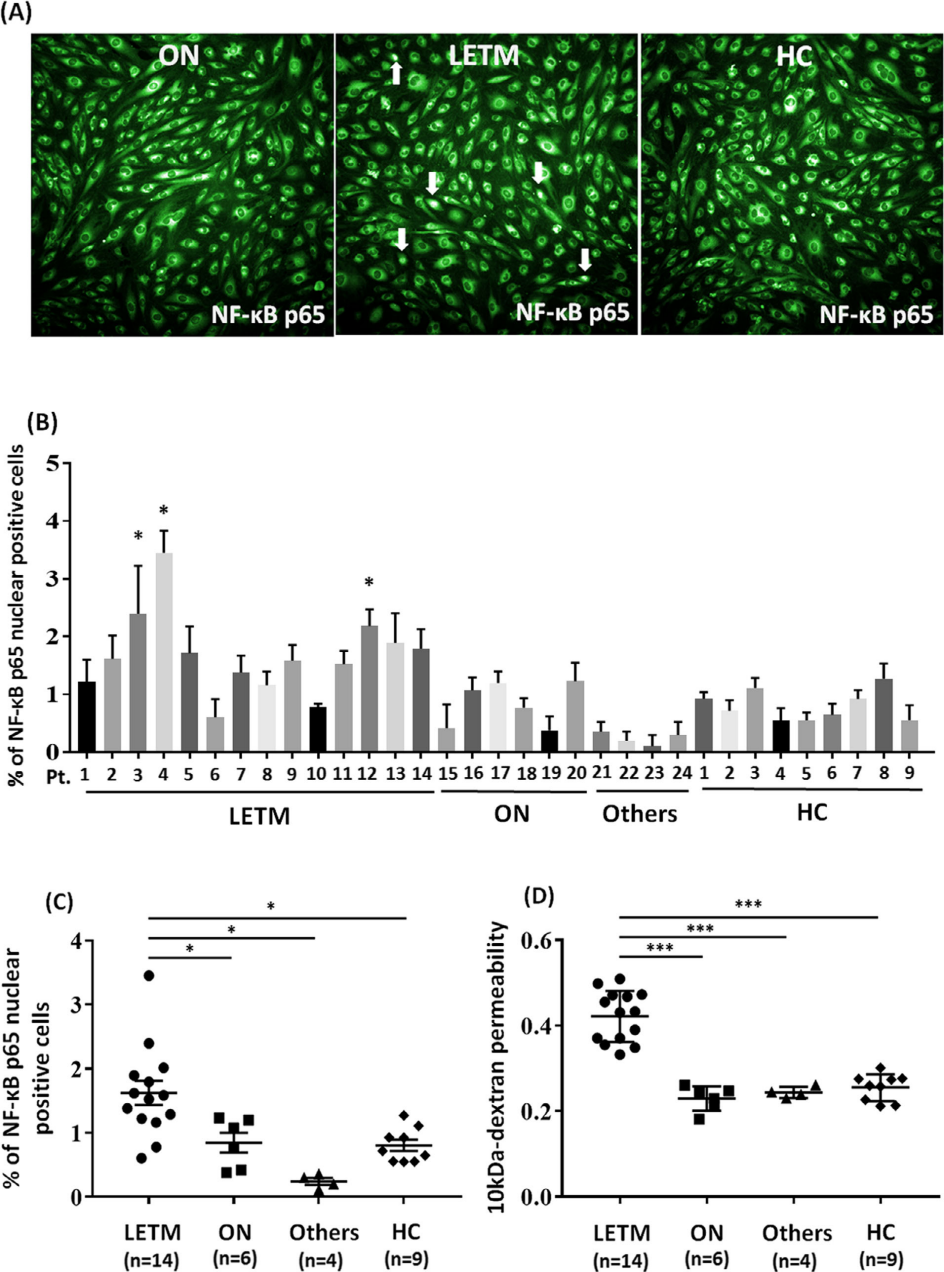
NF‐*κ*B p65 activation of brain endothelial cells after exposure to IgG from LETM or ON patients. (A) Immunostaining of human brain microvascular endothelial cells (TY10 cells) for NF‐*κ*B p65 (green) after exposure to IgG (500 μg/mL) from patients with longitudinally extensive transverse myelitis (LETM) or optic neuritis (ON), or healthy controls (HC). Images were captured by an In cell analyzer 2000 (B) Quantification of nuclear NF‐*κ*B p65‐positive TY10 cells by high‐content imaging after exposure to LETM‐IgG, ON‐IgG, other NMOSD phenotype (others)‐IgG, or control‐IgG (500 μg/mL). Data were normalized to cultures unexposed to human IgG and are shown as the mean ± SEM of four independent experiments performed in triplicate (*P < 0.05 vs. control followed by Tukey’s multiple comparison test). (C) Scatter plots of the number of nuclear NF‐*κ*B p65‐positive TY10 cells, as determined by high‐content imaging after exposure to LETM‐IgG (LETM group), ON‐IgG (ON group), other NMOSD phenotype‐IgG (others group), or control‐IgG (Healthy control group). The number of nuclear NF‐*κ*B p65‐positive cells in the LETM group was significantly increased in comparison to the ON, others and control groups. The P values were determined by a one‐way ANOVA followed by Tukey’s multiple comparison test (*P < 0.05 vs. control, ON or other group followed by Tukey’s multiple comparison test). (D) Scatter plots of the 10‐kDa dextran permeability of TY10 cells after exposure to LETM‐IgG, ON‐IgG, other‐IgG, or control‐IgG (***P < 0.001 vs. control, ON or other group followed by Tukey's multiple comparison test)

**Figure 2:**
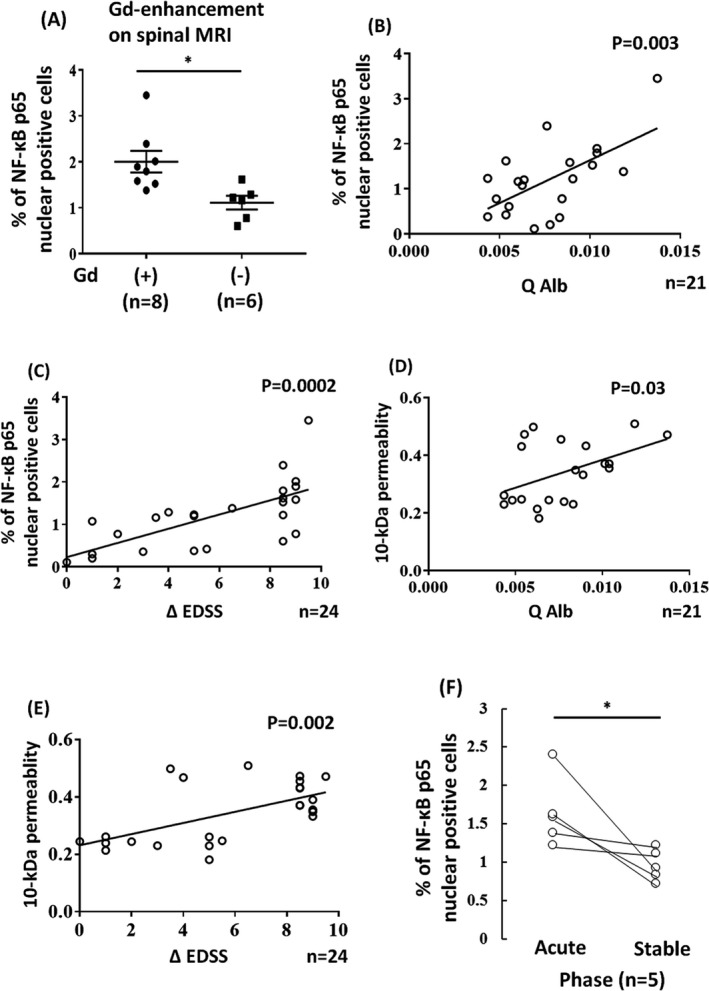
Correlations between the clinical findings and the percentage of NF‐*κ*B p65‐positive BMECs/BBB permeability after exposure to NMO‐IgG. (A) The number of nuclear NF‐*κ*B p65‐positive BMECs in the LETM patients with Gd‐enhanced lesions on spinal MRI was significantly increased in comparison to those without Gd‐enhanced lesions (*P < 0.05). (B and C) Correlation between the number of nuclear NF‐*κ*B p65‐positive cells after exposure to IgG from acute NMOSD patients and the albumin ratio (Q Alb) (B) and ΔEDSS (C). (D and E) The correlation between the 10‐kDa dextran permeability of TY10 cells after exposure to IgG from acute NMOSD patients and the albumin ratio (Q Alb) (D) and ΔEDSS (E). (F) The number of nuclear NF‐*κ*B p65‐positive cells was significantly decreased in the remission phase. Statistical significance was assessed by a paired two‐tailed *t*‐test (*P < 0.05)

Three IgGs (Patients 3, 4 and 12) from the LETM patients significantly induced the nuclear translocation of NF‐*κ*B p65 in BMECs in comparison those from ON patients and healthy controls (Fig. 1A and B). We next compared the percentage of nuclear NF‐*κ*B p65‐positive BMECs in the LETM, ON, other NMOSD phenotypes (others), and healthy control groups. The number of nuclear NF‐*κ*B p65‐positive cells in the LETM group was significantly increased in comparison to the ON, others, and healthy control groups (Fig. 1C). Furthermore, we confirmed that the permeability was increased after exposure to IgG from LETM patients but not after exposure to IgG from ON or other NMOSD patients or healthy controls (Fig. 1D). In addition, we determined the correlations between the spinal MRI and laboratory findings and the percentage of NF‐*κ*B p65‐positive BMECs after exposure to NMO‐IgG. LETM patients with Gd‐enhanced lesions in spinal MRI showed greater amount of NF‐*κ*B nuclear‐positive BMECs in comparison to those without Gd‐enhanced lesions (Fig. 2A). Higher percentage of NF‐*κ*B p65‐positive BMECs and higher permeability of BMECs incubated with NMO‐IgG was correlated with a higher Q Alb level and ΔEDSS (Fig. 2B–E). In addition, in the analysis using IgG obtained from the LETM patients between the acute and remission phases (in the same individual), the number of nuclear NF‐*κ*B p65‐positive cells was significantly decreased after exposure to IgG from patients during the remission phase (Fig. 2F).

**Figure 3:**
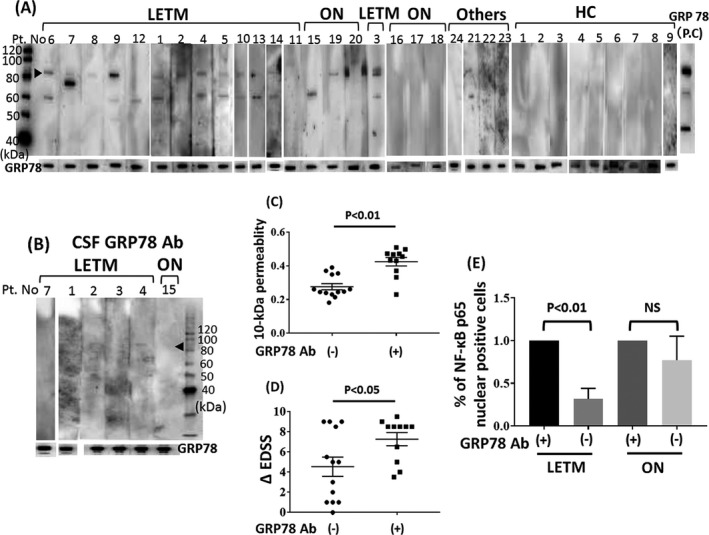
Western blotting of GRP78 autoantibodies in IgG from NMO patients. (A) The results of western blotting of individual IgG samples (5 μg/mL) from patients with LETM, ON, others and healthy volunteers, as determined using recombinant human GRP78 protein prepared from *Escherichia coli*. Arrowhead indicates an immunoreactive band corresponding to GRP78. Rabbit anti‐GRP78 antibodies were used as the positive control (P.C). (B) The presence of GRP78 antibodies in CSF samples from NMOSD patients (five LETM and one ON patient) according to a western blot analysis. Recombinant human GRP78 protein was used as the antigen. The arrowhead indicates an immunoreactive band corresponding to GRP78. (C) The 10‐kDa dextran permeability of TY10 cells in NMOSD patients with GRP78 antibody was higher than in those without these antibodies. (D) The increase in the ΔEDSS was correlated with the presence of GRP78 antibodies. (E) The effect of the removal of GRP78‐specific IgG from LETM‐IgG or ON‐IgG on the NF‐*κ*B p65 nuclear translocation in TY10 cells. Data are shown as the mean ± SEM of six independent experiments

A specific positive band against human GRP78 was detected in the IgG from NMOSD patients by western blotting using the recombinant protein prepared from *Escherichia coli*. The number of patients with GRP78 antibodies in the LETM group (10 of 14, 71%) was significantly higher in comparison to that in the ON group (1 of 6, 17%), the other NMDSD phenotype group (0 of 4, 0%) (Fig. 3A, Table 1). In contract, no bands were found in any of the serum samples from 10 healthy controls (Fig. 3A). The presence of CSF GRP78 antibodies was detected in only one LETM patient (patient 4) among six NMOSD patients (5 LETM and 1 ON patients; 1 of 6, 16%) (Fig. 3B). Positivity for GRP78 antibodies was significantly associated with an increased BBB permeability using our in vitro model (Fig. 3C) as well as with a higher ΔEDSS, a clinical marker of disease severity (Fig. 3D). The removal of GRP78 antibodies from LETM‐IgG, not ON‐IgG, resulted in less NF‐*κ*B nuclear translocation of BMECs (Fig. 3E).

## Discussion

It remains unclear why NMO predominantly affects the spinal cord and optic nerves. Some reports have shown that the optic nerve susceptibility of NMO patients may be associated with higher expression levels of AQP4 proteins and the relative abundance of large orthogonal arrays of particles that bind the anti‐AQP4 antibodies in astrocytic endfeet of the optic nerve in comparison to the brain.^3^, ^13^, ^14^ Another possible explanation is that dysfunction of the blood–optic nerve barrier (BONB) or blood–spinal cord barrier (BSCB) may determine the development of the clinical phenotype (ON or LETM), because this barrier restricts the entry of anti‐AQP4 antibodies into the optic nerve or spinal space. We recently reported that the GRP78 autoantibodies in NMO‐IgG were associated with the breakdown of the BBB in NMO.^10^ The aim of this study was to address the next question; whether BBB‐endothelial cell activation and GRP78 antibodies are correlated with the clinical phenotype and disease activity, and whether it is a clinical marker of the breakdown of the BBB in NMOSD.

The cell surface expression of GRP78 is involved in NF‐*κ*B signal transduction^15^ and the nuclear translocation of NF‐*κ*B p65 in BMECs, as a marker of BBB activation, is associated with BBB dysfunction.^10^ In the present study, we demonstrated that three IgGs from individual LETM patients significantly induced NF‐*κ*B p65 nuclear translocation in the BMECs in comparison to the IgGs from controls. As a group, we also observed the significant induction of cell activation and increase in BBB permeability in the LETM group than the ON, other NMOSD phenotype and healthy control groups. This effect was significantly decreased in the individual NMOSD patients during the remission phase. Furthermore, we found a significant correlation between endothelial cell activation in the BBB and two clinical markers of BBB dysfunction (Q Alb and Gd‐enhanced lesions)/clinical marker of disease severity (ΔEDSS) in NMOSD. A significant association with an increased BBB permeability and a higher Q Alb/ΔEDSS in NMOSD patients was also observed. Taken together, the activation of endothelial cells in the BBB, which was induced by NMO‐IgG, was associated with the LETM phenotype, the presence of clinical markers of BBB disruption, disease severity, and disease activity in NMOSD patients. Furthermore, the rate of GRP78 autoantibody positivity in the LETM group was significantly higher than that in ON group (LETM 71% vs ON 17%, others 0%). The presence of GRP78 antibodies was correlated with an increased ΔEDSS (disease severity) and BBB leakage in our in vitro BBB model. These results suggest that the phenotypical discrepancy between LETM and ON may be derived from the difference in the disruption of the BBB/BSCB that is caused by GRP78 autoantibodies. Namely, the large amount of GRP78 antibodies in IgG from definite NMO or LETM patients can cause the diffuse destruction of the BBB/BSCB and the massive entry of AQP4 antibodies, thus leading to the development of severe clinical symptoms and central nerve system (CNS) disability. In contrast, the lower amount of GRP78 autoantibodies in IgG from isolated ON patients is not enough to disrupt the BBB/BSCB; thus, these patients develop optic nerve symptoms without CNS symptoms.

Four LETM patients (patients 1, 2, 3, and 7) were positive for GRP78 antibodies in their serum samples but, not their CSF samples. The positivity of GRP78 antibodies in the CSF was less marked than that in the serum of NMOSD, suggesting that GRP78 antibodies may be produced by peripheral B cells. One LETM patient (patient 4) was positive for GRP78 antibodies in both the sera and CSF, possibly reflecting the entry of GRP78 antibodies into the CNS space due to BBB disruption. Interestingly, serum GRP78 antibodies were negative in one patient (patient 24), who developed only brainstem symptoms including area postrema, suggesting that anti‐AQP4 antibodies had entered the area postrema, where endothelial cells lack tight junctions and the AQP4 expression is enriched, leading to brainstem symptoms in this case.

The reason why LETM‐IgG but not ON‐IgG causes the breakdown of the BBB is not still clear. There are two possible explanations: LETM patients have a higher titer of GRP78 antibodies than ON patients. In the present study, the association between the GRP78 antibody titer and the NMO phenotype was not examined because we have not established an ELISA to determine the GRP78 antibody titer. The other possible explanation is the difference in the cell surface expression of GRP78 between BBB/BSCB‐endothelial cells and BONB‐endothelial cells. We found that the cell surface expression of GRP78 was abundant in BMECs.^10^ In contrast, the expression of GRP78 on BONB‐endothelial cells may be lower than that on BBB‐endothelial cells. Further studies using human BONB‐endothelial cells are needed to understand the role of GRP78 autoantibodies in the breakdown of the BONB.

## Author Contribution

FS and TK were responsible for conception and design of the study. FS, YH, YT, HN, YS, TM, SF, RS, and MH performed the experiments and evaluate the data. FS, HN, and TT were responsible for collecting sample and data from patients and preparing purified IgG. FS wrote the manuscript and TK edited the manuscript.

## Conflict of Interest

None.

## Patient Consent

Obtained.

## Ethic Approval

The study was approved by the ethics committees of Yamaguchi Universities.

## Provenance and Peer review

Not commissioned; externally peer‐reviewed.

## Supporting information


**Figure S1.** A NMOSD patient (Patient 24) presented with brainstem syndromes (double vision and dizziness), and a lesion of brainstem including area postrema was observed in the Magnetic Resonance Imaging (MRI).
