# Supplementary figures and images for: GRP 78 antibodies are associated with clinical phenotype in neuromyelitis optica
Source: Ann Clin Transl Neurol. 2019 Sep 30;6(10):2079–87. doi: 10.1002/acn3.50905 (PMC6801168; doi:10.1002/acn3.50905)

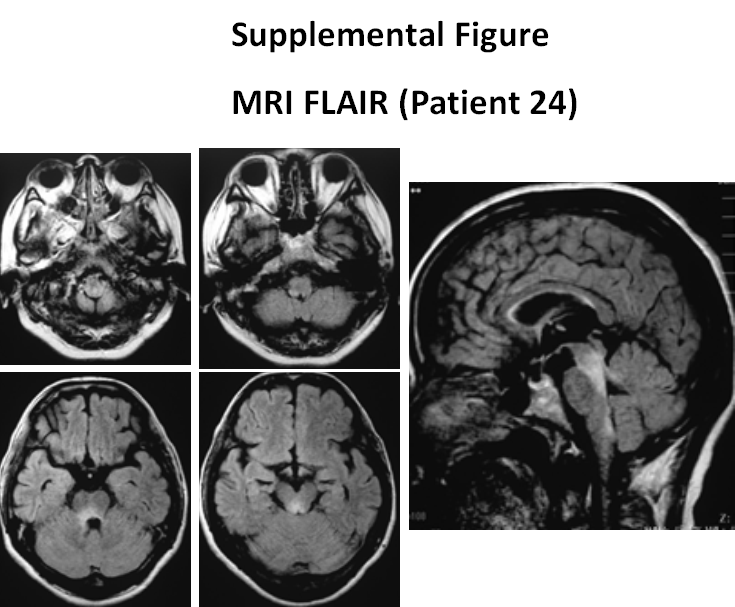

Supplement: Supplementary file 1 — Figure S1. A NMOSD patient (Patient 24) presented with brainstem syndromes (double vision and dizziness), and a lesion of brainstem including area postrema was observed in the Magnetic Resonance Imaging (MRI). [file ACN3-6-2079-s001.tif]
